# Humans Infected with Relapsing Fever Spirochete *Borrelia miyamotoi*, Russia

**DOI:** 10.3201/eid1710.101474

**Published:** 2011-10

**Authors:** Alexander E. Platonov, Ludmila S. Karan, Nadezhda M. Kolyasnikova, Natalya A. Makhneva, Marina G. Toporkova, Victor V. Maleev, Durland Fish, Peter J. Krause

**Affiliations:** Author affiliations: Central Research Institute of Epidemiology, Moscow, Russia (A.E. Platonov, L.S. Karan, N.M. Kolyasnikova, V.V. Maleev);; Municipal Clinical Hospital No. 33, Yekaterinburg, Russia (N.A. Makhneva, M.G. Toporkova);; Yale School of Public Health and Yale School of Medicine, New Haven, Connecticut, USA (D. Fish, P.J. Krause)

**Keywords:** Borrelia miyamotoi, human disease, relapsing fever, Borrelia burgdorferi, Borrelia garinii, bacteria, zoonoses, vector-borne infections, Russia, research

## Abstract

*Borrelia miyamotoi* is distantly related to *B. burgdorferi* and transmitted by the same hard-body tick species. We report 46 cases of *B. miyamotoi* infection in humans and compare the frequency and clinical manifestations of this infection with those caused by *B. garinii* and *B. burgdorferi* infection. All 46 patients lived in Russia and had influenza-like illness with fever as high as 39.5°C; relapsing febrile illness occurred in 5 (11%) and erythema migrans in 4 (9%). In Russia, the rate of *B. miyamotoi* infection in *Ixodes persulcatus* ticks was 1%–16%, similar to rates in *I. ricinus* ticks in western Europe and *I. scapularis* ticks in the United States. *B. miyamotoi* infection may cause relapsing fever and Lyme disease–like symptoms throughout the Holarctic region of the world because of the widespread prevalence of this pathogen in its ixodid tick vectors.

## Abstract 2

Disease may occur throughout the world because of the widespread prevalence of this pathogen in ixodid ticks.

*Borrelia miyamotoi*, discovered in Japan in 1995, belongs to the relapsing fever group of *Borrelia* (*1*). Relapsing fever borreliae infections are characterized by influenza-like illness and >1 relapse episode of bacteremia and fever. *B. miyamotoi* is more distantly related to *B. burgdorferi*, a group of spirochetes that includes *B. burgdorferi* s.l. strains (*B. afzelii*; *B. garinii*; and *B. burgdorferi* s.s., the causative agent of Lyme disease) (*2**,**3*). In Eurasia and North America, *B. miyamotoi* is found in a small percentage of all species of ixodid tick vectors of *B. burgdorferi*, including *Ixodes persulcatus* (*1**,**3**,**4*), *I. ricinus* (*5**–**7*), *I. scapularis* (*2**,**3**,**8**,**9*), and *I. pacificus* (*10*). It is transmitted transovarially and transstadially by ticks and coexists with *B. burgdorferi* (*2**,**3*). Recently, we discovered *B. miyamotoi* in *I. persulcatus* and *I. ricinus* ticks in the European and Asian regions of Russia. In these areas, human ixodid tick-borne infections, including those caused by *B. afzelii*, *B. garinii*, and viral tick-borne encephalitis virus (TBEV; genus *Flavivirus*) are endemic and transmitted by the same tick species.

Despite the presence of *B. miyamotoi* in vector ticks, to our knowledge, human disease caused by this spirochete has not been definitively established. We previously noted presumptive *B. miyamotoi* infection in residents of central Russia with influenza-like illness but were uncertain whether their clinical manifestations were caused by co-infecting *B. burgdorferi* s.l. species (*11**–**13*). To confirm those findings and develop initial estimates of the prevalence and severity of *B. miyamotoi* infection, we conducted a comparative cohort study. We used improved antibody assays and PCRs to compare the relative frequency and clinical manifestations of *B. miyamotoi* infection with those of *B. garinii* infection in Russia and *B. burgdorferi* infection in the United States.

## Methods

### Study Design

We enrolled patients admitted to Municipal Clinical Hospital No. 33 in Yekaterinburg City, Russia, from May 19 through August 25, 2009, for suspected tick-borne infection. Yekaterinburg is in the Asian part of Russia, ≈1,200 miles east of Moscow. Viral tick-borne encephalitis and acute borreliosis are highly endemic to this region. Patients with moderate or severe disease are usually hospitalized.

We compared the clinical characteristics of patients experiencing laboratory-confirmed *B. miyamotoi* infection with those of patients experiencing *B. garinii* infections from the same area and with those of patients who experienced *B. burgdorferi* infection in the northeastern United States. The US data came from a tick-borne diseases study conducted during 1991–2008 (*14**,**15*). For each patient at all study sites, we recorded the presence or absence of a standard set of 11 clinical manifestations. All patients signed an informed consent form in accordance with the institutional review boards of the Municipal Clinical Hospital in Yekaterinburg City or the University of Connecticut School of Medicine.

**Figure 1:**
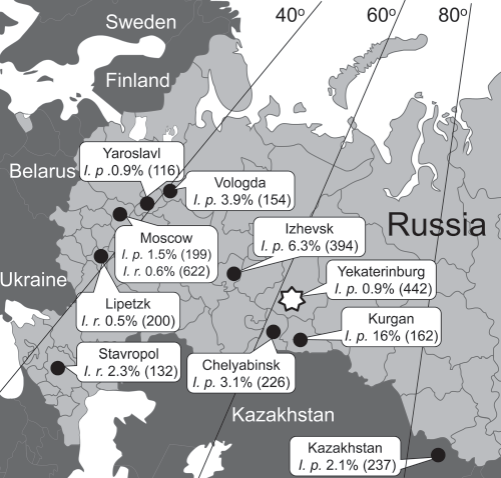
Percentage of *Ixodes persulcatus* (*I. p.*) and *I. ricinus* (*I. r.*) ticks infected with *Borrelia miyamotoi* in Russia. The number of ticks that were tested is given in parenthesis. Star indicates study location of human *B. miyamotoi* infection.

We also determined the frequency of *B. garinii*, *B. afzelii*, *B. burgdorferi*, and *B. miyamotoi* in *I. persulcatus* and *I. ricinus* ticks in Yekaterinburg and several additional regions of Russia (Figure 1). Ticks were collected by drag cloth, visually identified to species level, and analyzed by PCR to identify specific *Borrelia* species.

### Case Definitions

Diagnosis of *B. miyamotoi* infection required the report of a tick bite, presence of clinical manifestations consistent with borreliosis, and laboratory evidence of *B. miyamotoi* infection. Clinical manifestations included fever, headache, chills, fatigue, vomiting, and myalgia. Confirmation of active infection consisted of amplification of *B. miyamotoi* DNA/RNA in blood by species-specific PCR and detection of anti-borreliae immunoglobulin (Ig) M in acute- and/or convalescent-phase serum samples.

In Russia, diagnosis of *B. garinii* infection required report of a tick bite, physician diagnosis of erythema migrans (EM; an expanding, ring-like erythematous rash >5 cm in diameter), or an influenza-like illness. Confirmation of infection consisted of amplification of *B. garinii* DNA/RNA in blood by specific PCR, followed by direct sequencing of 5S-23S ribosomal RNA (rRNA) intergenic spacers, and detection of anti-borreliae IgM in acute- and/or convalescent-phase serum samples.

In the United States, diagnosis of *B. burgdorferi* infection required a physician’s diagnosis of EM or an influenza-like illness. For all cases, confirmation of infection consisted of a >4-fold increase in anti–*B. burgdorferi* antibody in acute- and convalescent-phase serum samples. The diagnosis of TBEV infection was based on a viral-like illness, including headache (with or without meningitis or encephalitis), amplification of TBEV RNA in blood by species-specific PCR, and/or detection of anti-TBEV IgM in an acute-phase serum sample.

### Laboratory Assays

#### PCR

The PCR we used enabled detection of DNA and RNA sequences. DNA/RNA was extracted from 2 mL of whole venous blood with EDTA or from tick suspensions by using an AmpliSens Riboprep Kit (Central Institute of Epidemiology, Moscow, Russia) according to the manufacturer’s instructions. Of the blood samples used for PCR, 81% were obtained at the time of hospital admission and 96% within 2 days of admission. To assay the inhibitory effect of blood and tick extracts on the PCR, all samples were spiked with a universal RNA recombinant control having a known number of RNA copies per milliliter. Reverse transcription of RNA to cDNA was performed by using an Amplisens Reverta-L Kit (Central Institute of Epidemiology). The cDNA samples were assayed for *B. miyamotoi* and other tick-borne pathogens by using real-time quantitative PCR (qPCR) assays in a Rotor Gene 6000 cycler (Corbett Life Science, Concorde, New South Wales, Australia).

The cDNA samples were divided into 2 aliquots, and different types of real-time qPCR were performed on each. The first used in-house primers and a probe that targeted the 16S rRNA gene of *B. miyamotoi.* The inclusion of the reverse transcription procedure improved the detection sensitivity because the 16S rRNA that also became detectable is present in higher copy numbers than the 16S rRNA gene. The detection limit of at least 5 × 10^3^ copies/mL was determined by using positive recombinant DNA of the *B. miyamotoi* 16S rRNA gene fragment with a known number of copies. The *B. miyamotoi*–specific forward and reverse primers at 360 nmol/L were, respectively, Brm1 5′-CGCTGTAAACGATGCACACTTGGTGTTAATC-3′ and Brm2 5′-CGGCAGTCTCGTCTGAGTCCCCATCT-3′. The corresponding dye-labeled probe (final concentration 100 nmol/L) was R6G-CCTGGGGAGTATGTTCGCAAGAATGAAACTC-BQH1. The PCR conditions were 95°C for 15 min; followed by 10 cycles at 95°C for 20 s, 67°C for 50 s, and 72°C for 20 s; then by 40 cycles at 95°C for 20 s, 60°C for 50 s, and 72°C for 20 s. The fluorescence signal was recorded at the 60°C step for the last 40 cycles. Each run included negative controls and positive recombinant control DNA of the *B. miyamotoi* 16S rRNA gene fragment as a standard. PCR-based detection of *B. burgdorferi* s.l., *Anaplasma phagocytophilum*, *Ehrlichia chaffeensis, Ehrlichia muris*, and TBEV was performed on the second cDNA aliquot by using a commercial multiplex PCR (AmpliSens TBEV, *B. burgdorferi* s.l., *A. phagocytophillum, E. chaffeensis/E. muris*-FL; Central Institute of Epidemiology) (*16*), according to the manufacturer’s instructions. This assay was designed to detect, but not discriminate between, *B. afzelii*, *B. burgdorferi* s.s., and *B. garinii*. The same assays were used to detect specific DNA/RNA in ticks and humans.

The specificity of *B. miyamotoi* and *B. burgdorferi* s.l. assays was confirmed by direct sequencing of flagellin gene fragments and/or 16S rRNA gene fragments and/or 5S-23S rRNA intergenic spacer amplified from blood samples of the same patients or from the same ticks (GenBank accession nos. GU797331–GU797350, JF951378–JF951392). Of the 97 borreliae sequenced, results of DNA amplification using species-specific PCR were entirely consistent with the sequencing results. Absence of false-positive PCR results means that our PCRs were highly specific.

Amplification and further direct sequencing of the *B. miyamotoi* flagellin gene were performed by using degenerate primers FLA120F 5′-AGAATTAATMGHGCWTCTGATGATG-3′ and FLA920R 5′-TGCYACAAYHTCATCTGTCATT-3′ (*2**,**5*). The 16S rRNA gene fragment was amplified and sequenced by using 2 primers pairs: first Bf1 5′-GCTGGCAGTGCGTCTTAAGC-3′ and Brsp2 5′-CCTTACACCAGGAATTCTAACTTCCYCTAT-3′, second Brsp1 5′-GGGGTAAGAGCCTACCAAGGCTATGATAA-3′ and Br1 5′-GCTTCGGGTACTCTCAACTC-3′ (*5*). Borrelial 5S-23S rRNA intergenic spacer was amplified and sequenced by using nested PCR with outer primers pairs IGSa 5′-CGACCTTCTTCGCCTTAAAGC-3′ and IGSb 5′-AGCTCTTATTCGCTGATGGTA-3′ and inner primers pair IGSe 5′-CCTTAAAGCTCCTAGGCATTCACCA-3′ and IGSd 5′-CGCGGGAGAGTARGTTATTGCGA-3′ (*17*). Nucleotide sequences were aligned, compared, and analyzed by using MEGA4.1 (www.megasoftware.net), ClustalW (www.clustal.org), and BLAST (www.ncbi.nlm.nih.gov/blast/Blast.cgi).

#### ELISA

Serum samples collected at the time of admission and 1–2 weeks later were tested for anti-borrelial IgM and IgG. Serologic evidence of exposure to borreliae was detected by ELISA EUROIMMUN EI 2132–9601 M and EI 2132–9601–2 G (EUROIMMUN AG, Lübeck, Germany). The ELISA consisted of a mixture of whole antigens from *B. afzelii*, *B. burgdorferi*, and *B. garinii* and thus could detect but not discriminate specific antibody against any of these species. Seroconversion in patients infected with the relapsing fever borrelia *B. persica* also has been detected by EUROIMMUN assay (*18*). Serum from most *B. miyamotoi–*positive patients reacted to the antigen(s) in this assay. Anti-TBE IgM was detected by the semiquantitative EUROIMMUN ELISA EI 2661–9601 M.

### Statistical Analyses

Comparisons were performed by using the Mann-Whitney *U* test (independent numeric interval variables), χ^2^ test (categorical variables), and corresponding exact tests, if necessary; p<0.01 was statistically significant. Data were analyzed by using SPSS version 11.0.1 (SPSS Inc., Chicago, IL, USA).

## Results

### Study Population

Among 302 patients evaluated for presumptive tick-borne infection, *B. miyamotoi* infection was found for 51 (17%) (Table 1). Of these 51 patients, anti-borrelial IgM was found at the time of admission for 6, anti-borrelial IgM seroconversion was demonstrated 2 weeks after admission for 40, no such antibody was found for 2, and laboratory evidence of TBEV co-infection was found for 3. Only the 46 patients who had amplifiable *B. miyamotoi* DNA and anti-borrelial IgM and who were not coinfected with TBEV were included in further analyses. For 18 of these patients, anti-borrelial IgG was absent at admission but detected 2 weeks after admission; subsequent IgG testing was not performed because all patients had IgM by 2 weeks. Attempts to detect *B. miyamotoi* on standard Giemsa-stained blood smears and in culture in 6 patients yielded negative results. These tests were performed 4–6 days after onset of illness but before initiation of antimicrobial drug therapy.

**Table 1:** Classification of suspected tick-borne infections, Yekaterinburg City, Russia, May–August 2009*

Classification	Total no. patients	No. patients with erythema migrans	Amplifiable DNA/RNA, by PCR				Antibody	
			*Borrelia miyamotoi*	*B. burgdorferi* s.l.	TBEV		*Borrelia* IgM	TBEV IgM
*B. miyamotoi* infection, confirmed	46	4	46	0	0		46	0
*B. miyamotoi* infection, unconfirmed	2	0	2	0	0		0	0
*B. miyamotoi* infection, TBEV co-infection	3	0	3	0	0		2	3
*B. garinii* infection, confirmed	21	19	0	21	0		21	0
*B. burgdorferi* s.l. infection	83	83	0	0	0		59	0
*Borrelia* spp. infection, unconfirmed	42	0	0	0	0		42	0
TBEV infection, confirmed	21	0	0	0	5		0	21
TBEV, *B. burgdorferi* s.l. co-infection	9	9	0	0	2		ND	9
TBEV, *Borrelia* spp. co-infection	11	0	0	0	3		11	11
Other inflammatory disease	64	0	0	0	0		0	0

*TBEV, tick-borne encephalitis virus; Ig, immunoglobulin; ND, not determined.

*B. garinii* infection was diagnosed for 21 (7%) patients, all of whom had amplifiable *B. garinii* DNA/RNA (GenBank accession nos. GU797347, GU797348, GU797349, GU797350) and anti-borreliae IgM in their blood. EM was found on all but 2 *B. garinii*–infected patients.

Of the remaining 230 patients, 83 had apparent *B. burgdorferi* s.l. infection (59 had EM and anti-borreliae IgM in acute- and/or convalescent-phase serum, and 24 had EM alone); 42 had unconfirmed *Borrelia* spp. infections with anti-borreliae IgM but lacked EM and were *Borrelia* spp. negative on PCR; 41 had TBE; 37 had fever of unknown origin after tick bite; and 27 had other diagnoses, including enteroviral infection, mononucleosis, or pyelonephritis. None of the 302 patients had any PCR-based evidence of *B. afzelii*, *A. phagocytophillum,* or *E. muris* infection.

### Clinical Manifestations

Patients from Russia with *B. miyamotoi* and *B. garinii* infection and patients from the United States with *B. burgdorferi* infection were similar in age and sex. Time from tick bite to onset of symptoms was longer and time from symptom onset to hospital admission was shorter for *B. miyamotoi* patients than for *B. garinii* patients (Table 2).

**Table 2:** Patient characteristics and infection timeline for *Borrelia* spp. infections, by species*

*Borrelia* species	No. patients infected				Infection timeline, median no. days (IQR)	
		Patient characteristics			Tick bite to symptom onset	Symptom onset to hospital admission
		Median age, y (range)	Male sex, no. (%)			
*B. miyamotoi*	46	54 (21–77)	24 (52)		15 (12–16)	1 (1–2)
*B. garinii*	21	58 (18–87)	11 (52)		10 (7–13)†	5 (2–9)†
*B. burgdorferi*	92	50 (14–79)	49 (53)		NA	NA

*IQR, interquartile range; NA, not available. †p<0.001 in comparison with patients with *B. miyamotoi* infection.

More systemic manifestations, including fever and headache, were reported for *B. miyamotoi* patients than for *B. garinii* and *B. burgdorferi* patients (Table 3). Maximum temperatures measured at home and in the hospital were higher for *B. miyamotoi* patients (39.0°C, interquartile range [IQR] 38.8–39.5°C) than for *B. garinii* patients (37.6°C, IQR 38.8–39.5°C; p<0.001). Duration of fever was relatively short and did not differ significantly for *B. miyamotoi* and *B. garinii* patients (3.4 ± 1.4 and 3.3 ± 2.8 days, respectively). Body temperature began to return to reference range before antimicrobial drug therapy was initiated, as has been described for relapsing fever patients, in all but 1 *B. miyamotoi* patient. Hospital stay was longer for *B. miyamotoi* patients (median 20 days, IQR 15–22 days) than for *B. garinii* patients (median 10 days, IQR 10–13 days; p<0.001).

**Table 3:** Clinical manifestations in patients with *Borrelia* spp. infection, Yekaterinburg City, Russia, 2009, and northeastern United States, 1991–2008*

Manifestation	% Patients				p value		
	*B. miyamotoi*, n = 46	*B. garinii,* n = 21	*B. burgdorferi*, n = 92		*B. miyamotoi* vs. *B. garinii*	*B. miyamotoi* vs. *B. burgdorferi*	*B. garinii* vs. *B. burgdorferi*
Individual							
EM	9	91	89		<0.001	<0.001	>0.99
Multiple EM	0	14	7		0.03	0.18	0.36
Fever†	98	67	32		0.001	<0.001	0.005
Fatigue	98	86	74		0.09	<0.001	0.4
Headache	89	57	63		0.007	0.001	0.63
Chills	35	10	43		0.04	0.36	0.005
Myalgia	59	52	63		0.8	0.71	0.46
Arthralgia	28	29	62		>0.99	<0.001	0.007
Nausea	30	10	24		0.07	0.42	0.24
Vomiting	7	5	7		>0.99	>0.99	>0.99
Neck stiffness	2	0	38		>0.99	<0.001	<0.001
Overall							
No. symptoms, mean ± SD	4.5 ± 1.4	4.2 ± 2.0	5.0 ± 2.3		0.43	0.13	0.13
No. symptoms (excluding EM and multiple EM), mean ± SD	4.5 ± 1.4	3.1 ± 1.9	4.1 ± 2.3		0.007	0.46	0.09

*EM, erythema migrans. †Maximum axillary temperature >37.2°C for patients in Russia and maximum oral temperature >37.7°C for patients in the United States.

Although mean peripheral leukocyte and platelet counts were lower for patients with *B. miyamotoi* than *B. garinii* infection, they were within the reference range. Proteinuria and transient elevation of serum alanine aminotransferase and aspartate aminotransferase concentrations were found for 3× more *B. miyamotoi* patients than *B. garinii* patients (51% and 68% vs. 15% and 20%, respectively, p<0.01), but no nephritis or hepatitis was clinically apparent. We found similar clinical and laboratory results when we omitted from analysis the 4 *B. miyamotoi* patients with EM who might have been co-infected with *B. burgdorferi* s.l.

### Therapy and Clinical Outcome

Antimicrobial drug therapy for the *B. miyamotoi* patients was started ≈1 week after admission when IgM-based serologic tests results confirmed the diagnosis (median 7 days, IQR 6–10 days). Therapy consisted of ceftriaxone, 2 g intravenously every 24 hours for 2 weeks (42 patients) or doxycycline 100 mg orally every 12 hours for 2 weeks (2 patients). Two patients received no antimicrobial drug while hospitalized; 1 later received doxycycline at home, and the other was readmitted to the hospital for relapse and received ceftriaxone. Patients with *B. garinii* infection received doxycycline (71%) or ceftriaxone (29%) immediately after admission because diagnosis of borreliosis, based on presence of EM, was made at the time of admission. *B. burgdorferi* patients all received doxycycline, 100 mg orally every 12 hours, or amoxicillin, 500 mg orally every 8 hours, for 2–4 weeks. A Jarisch-Herxheimer reaction was noted for 7 (15%) of the 46 *B. miyamotoi* patients. More such reactions might have been expected if treatment had not been delayed until ≈1 week after admission. A single course of ceftriaxone or doxycycline appeared to clear *B. miyamotoi* infection.

### Relapsing Infection

**Figure 2:**
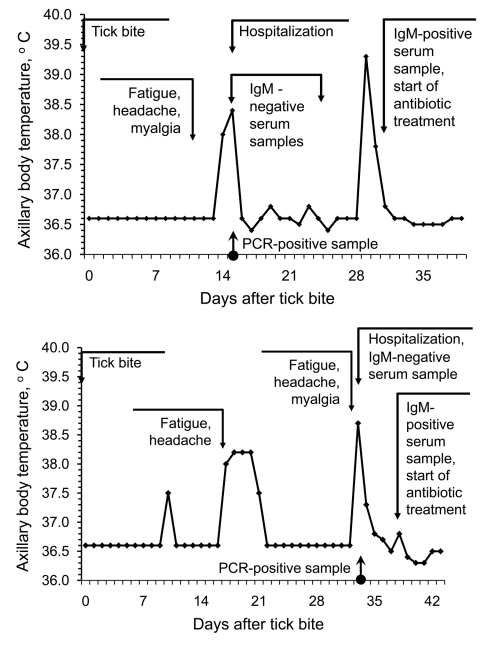
Examples of relapsing fever episodes in 2 patients with *Borrelia miyamotoi* infection. Arrows indicate the timing of tick bite, hospital admission, PCR testing, anti-borreliae immunoglobulin (Ig) M testing, and initiation of antimicrobial drug therapy.

Of the 46 *B. miyamotoi* patients, 5 (11%, 95% confidence interval 2%–20%) experienced relapse of febrile illness; 1 patient experienced 2 relapses before hospital admission, and 4 experienced 1 relapse after hospitalization but before the start of antimicrobial drug therapy. Thus, antimicrobial drugs might have prevented relapse in those who received this therapy. The mean time between relapses was 9 days (range 2 days to 2 weeks). The maximum fever and duration of illness did not differ significantly for the first and second episodes of illness (Figure 2). No clinical or laboratory findings indicated other infections (including blood-borne, skin, neurologic, respiratory, cardiac, gastrointestinal, and urologic) or medical conditions that could account for these febrile episodes.

### *B. miyamotoi* in Ticks

During 2004–2007, we found *B. miyamotoi*–infected *I. persulcatus* ticks in the regions where the human cases had been noted (*11**,**13*), namely, 0.9% of 442 ticks in Yekaterinburg and 6.4% of 394 ticks in Izhevsk. *B. miyamotoi–*infected *I. persulcatus* and *I. ricinus* ticks were found in regions where human cases have not yet been identified (Figure 1). These findings were confirmed by direct sequencing of PCR DNA amplification products (GenBank accession nos. GU797336, GU797337, GU797338, GU797346, JF951378–JF951392).

### Genetic Characteristics of *B. miyamotoi*

**Figure 3:**
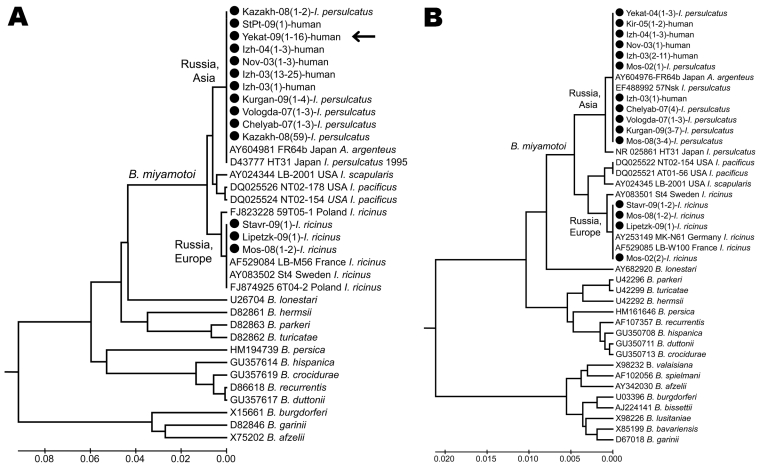
Phylogenetic tree of *Borrelia* spp. detected in persons and ticks, based on flagellin gene fragment (A) and16S rRNA gene fragment (B). Sequences were aligned and analyzed by using MEGA4.1 software (www.megasoftware.net). Genetic trees were constructed from the partial nucleotide sequences of the flagellin gene and the 16S rRNA gene by using the Kimura 2-parameter model and the unweighted pair group method with arithmetic mean. Arrow indicates the 16 *Borrelia* spp. from Yekaterinburg in 2009 that had the same nucleotide sequence. Circles indicate sequences that we listed in GenBank (accession nos. GU797331–GU797346 and JF951378–JF951392). Sequences for *B. burgdorferi* sensu lato and relapsing fever borreliae are shown for comparison. Scale bars indicate genetic distance.

The nucleotide sequences of 16S rRNA and flagellin gene fragments of all *B. miyamotoi* isolates from humans and *I. persulcatus* ticks were almost indistinguishable from the corresponding sequences of the prototype Japan HT-31 strain (*1*) (Figure 3). *B. miyamotoi* from *I. ricinus* ticks collected in the European part of Russia were more closely related to European *B. miyamotoi* strains (*5**,**6*).

## Discussion

We provide confirmatory evidence of *B. miyamotoi* infection in humans. Most patients experienced clinical manifestations similar to those caused by *B. burgdorferi* s.l. and relapsing fever *Borrelia* infections, a finding consistent with the genetic characteristics of this novel spirochete. Febrile relapses occurred in only 1 of 10 *B. miyamotoi* patients, 2 days to 1 month after the initial illness; however, early treatment may have prevented subsequent relapse for other patients. Although the febrile episodes at home might have been caused by other illnesses, the onset of fever within 2 weeks after a tick bite was consistent with the incubation period of infection with borreliae. Furthermore, no clinical or laboratory findings indicated other infections or medical conditions that could account for the febrile episodes. EM was found in ≈1 of 10 *B. miyamotoi* patients, but these patients might have had unrecognized *B. burgdorferi* s.l. co-infection. A single course of ceftriaxone or doxycycline seemed to clear *B. miyamotoi* infection. Although effective therapy is available, appropriate diagnosis and therapy are complicated by lack of awareness of *B. miyamotoi* as a human pathogen, the nonspecific symptoms of infection, and the absence of standardized and widely available assays. We found no PCR-based evidence of infection caused by *B. afzelii*, *A. phagocytophilum,* or *E.muris* in the patients, although these pathogens have been detected in *Ixodes* spp. ticks in the same region (*16*). There is only anecdotal evidence of *B. afzelii* infection confirmed by culture or PCR in Russia and none in the Yekaterinburg region. Relapsing fever borreliae other than *B. miyamotoi* were not found in Yekaterinburg.

*B. miyamotoi* infection may cause substantial health problems in the regions of Russia where it has been found, given its relatively high incidence and associated severity of disease. On the basis of the number of patients with *B. miyamotoi* infection in Yekaterinburg Hospital in 2009 and the populations of Yekaterinburg Province (4,395,000), we estimate that the minimal incidence of *B. miyamotoi* infections is 1 case per 100,000 population. According to official federal notification, during the past 10 years ≈8,000 cases of human borreliosis occur in Russia annually (*12*). *B. miyamotoi* infection seems to constitute at least 1/4 of all clinical tick-borne borreliosis cases in Yekaterinburg. If other *Borrelia* spp.–endemic areas have a similar rate of *B. miyamotoi* infection as Yekaterinburg (and our tick data suggest that this assumption is reasonable), >1,000 *B. miyamotoi* cases might occur in Russia each year. More studies are necessary to determine if this projection is accurate.

Acute *B. miyamotoi* infection was more severe than early stage *B. burgdorferi* infection. The time from symptom onset to hospital admission was shorter, and the number of clinical manifestations was greater for patients with *B. miyamotoi* infection than with *B. garinii* infection. Relapsing febrile episodes were only reported for *B. miyamotoi* patients. Such multiple disease episodes not only have an adverse effect on a patient’s health but also may result in costly medical bills, many days or weeks of lost wages, and medical misdiagnosis (*19**–**22*). Co-infection of *B. miyamotoi* with other ixodid tick–transmitted agents may increase disease severity (*15**,**23*). Additional problems that might occur with *B. miyamotoi* infection are ocular, neurologic, respiratory, cardiac, and pregnancy complications associated with relapsing fever (*19**–**22*).

Our study had several limitations. Attempts to detect *B. miyamotoi* on blood smear or in culture were not successful, although we confirmed *B. miyamotoi* infection with a combination of qPCR, genetic sequencing, clinical, and seroconversion evidence. The comparison of clinical manifestations of *Borrelia* spp. infection of patients from Russia and the United States was complicated by enrollment at different times and from different locations, although we assessed the same 11 clinical manifestations at each location. The possibility that the clinical description of our *B. miyamotoi* cases was compromised by unrecognized co-infection with *B. burgdorferi* s.l. is unlikely. The expected number of cases of co-infection depends on the prevalence of the pathogens in ticks in the region (*3**,**11**,**24*), and this number is even fewer than the 4 *B. miyamotoi* patients with EM we found. Inclusion or exclusion of these 4 cases had no effect on our comparative analysis with patients who did not have *B. miyamotoi* infection. We limited our description of *B. garinii* cases to those that were confirmed by detection of amplifiable *B. garinii* DNA/RNA, although such cases may be more severe than those in which such DNA/RNA cannot be detected (*25**,**26*). Patients with *B. burgdorferi* s.l. PCR–negative results experienced fewer symptoms and milder fever than did patients with *B. burgdorferi* s.l. PCR–positive results. Our analysis of patients with *B. miyamotoi* and *B. garinii* infection was limited to those who were hospitalized, although hospital admission policy in these regions of Russia is liberal because of concern about TBE and problems associated with *B. burgdorferi* infection.

The geographic dispersion and extent of *B. miyamotoi* disease in humans are unclear, but the infection probably occurs outside of Russia, given the comparative infection rates of vector ticks in Russia and at several locations in Europe and the United States (*2**–**8*). In the northeastern United States, ≈15% of all spirochetes carried by *I. scapularis* ticks are *B. miyamotoi* (*2*). Cases may remain undiagnosed because of the nonspecific nature of the illness, which might be confused with viral infections or such tick-borne infections as Lyme disease, babesiosis, anaplasmosis, or ehrlichiosis, and because of the lack of laboratory tests for confirmatory diagnosis (*19**–**22*).

*B. miyamotoi* infection may have negative health consequences, including relapsing disease that may last for months and may not respond to inappropriate antimicrobial drug therapy. The discovery of a *Borrelia* sp. that is pathogenic in humans and transmitted by an array of ixodid ticks greatly expands the potential geographic distribution of this disease (*1**–**11*). Further investigation of possible *B. miyamotoi* infection in humans is warranted wherever *I. pacificus, I. persulcatus, I. ricinus*, and *I. scapularis* ticks are found.
